# Multifocal periapical cemental dysplasia in periodontal Ehlers–Danlos syndrome combined with leukoencephalopathy in the mutation of c.890G > a, G297D [pEDS]

**DOI:** 10.1002/ccr3.6490

**Published:** 2022-11-04

**Authors:** Manfred Nilius, Minou Helene Nilius, Charlotte Müller, Guenter Lauer, Koch Berit, Kohlhaas Marcus

**Affiliations:** ^1^ Niliusklinik Dortmund Germany; ^2^ Department of Oral and Maxillofacial Surgery University Hospital "Carl Gustav Carus", Technische Universität Dresden Dresden Germany; ^3^ Department of Internal Medicine, Cardiology, Preveo‐Center Dortmund Germany; ^4^ Department of Ophthamology, ST.‐Johannes‐Hospital Dortmund Germany

**Keywords:** cone‐beam, densitometry, disease, dysplasia, ehlers‐danlos syndromes, jaw, leukoencephalopathy, oral manifestation, periapical

## Abstract

Periodontal Ehlers‐Danlos syndrome (pEDS) is a rare disorder caused by heterozygous mutations in complement 1 subunit genes C1R and C1S. To date, 148 cases have been described in the literature.We describe a case of a suspected de novo‐mutation of pEDS with generalized Periapical cemental dysplasia (PCD) and cerebral leukoencephalopathy.

## Graphical abstract

We describe a case of pEDS with moderate dentition but generalized periapical cemental dysplasia as a possible late sequela and additional cerebral leukoencephalopathy.

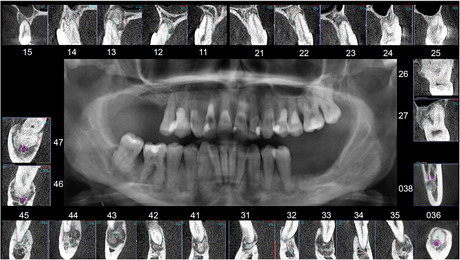

## 1 INTRODUCTION

Periodontal Ehlers–Danlos syndrome (pEDS; formerly: EDS type VIII) is a rare disorder caused by heterozygous mutations in complement 1 subunit genes C1R and C1S.^1^ To date, 148 cases have been described in the literature.^2^

The diagnosis is based on major clinical criteria, including periodontitis with early‐onset, persistent pretibial plaques, easy bruising, and a lack of attached gingiva. The minor clinical criteria are joint hypermobility, skin hyperextensibility and fragility, abnormal scarring, and increased rate of infections, hernia, and marfanoid facial features. These criteria consolidate the diagnosis. As a pathognomonic feature, a lack of attached gingiva is assumed.^3^

Periapical cemental dysplasia (PCD) is considered a non‐neoplastic proliferation of fibrous tissues and cementum‐like hard tissues.^4^, ^5^, ^6^ PCD is characterized by the presence of vital pulp and is often accidentally discovered by the dentist during a general radiographic survey that presents multiple sclerotic masses affecting the cancellous portion of the jaw and tooth‐bearing areas without any clinical signs.^7^ PCD is rarely seen in all four quadrants and, to the best of the authors' knowledge, has not been described as occurring in pEDS. We describe a case of a suspected de novo‐mutation of pEDS with generalized PCD and cerebral leukoencephalopathy.

## 2 REPORT

A 39‐year‐old woman visited our clinic with painless swelling of the gums in the area of the upper right canine and anterior mandible. In addition, the patient reported loosened teeth.

The patient was the first and only child after three miscarriages of the mother. The patient's father died early of gastric cancer with osseous involvement. However, he had problems with his teeth from the age of 10 years and tooth loss from the age of 30 years as well as a retinal detachment. The patient's mother and seven siblings of the father were interviewed with regard to pEDS typical symptoms and denied having noticed any symptoms in themselves and the father. In addition, molecular genetic testing was performed on all relatives with no evidence of pEDS.

The patient noticed easy bruising following slight trauma since childhood and brown pretibial hematomas with atrophic scars. She reported an increased growth period during her time in school and was the tallest in the elementary school class, even before puberty. She had an early onset of gingivitis and discolorations of the teeth. Uterus bicornae were confirmed gynecologically. A dermatological consultation revealed hyperextensibility of the skin. Other extraoral manifestations were a marfanoid habit with a body mass index of 21, hypermobility, and generalized hyperextensibility of the joints with a Beighton score of 5/9.^8^ Doppler‐sonography detected slight cardiovascular abnormalities showing a prolapsus of the dorsal tricuspid valve with minimal reflux and without rheological significance. The patient reported a recurrent temporary visual impairment. The ophthalmological examinations, however, showed normal anatomical and physiological findings. In particular, Brittle cornea syndrome (BDS) was not found.

Intraoral findings: The patient reported tooth loss of the upper right and lower left molars at 12–14 years of age due to previous orthodontic treatment. We tested the positive vitality of all the remaining teeth. The gingival and periodontal indices utilized were the Community Periodontal Index of Treatment Needs (CPI‐TN) (WHO)^9^: Grade 2^9^ and the Gingival Bleeding Index (GBI): 13%.^10^ The oral biofilm (Micro‐Ident, Hain‐Lifescience, Nehren, Germany) revealed moderate concentrations of *Treponema denticola* (x < 10^5^) and *Tannerella forsythia* (x < 10^4^). No *Aggregatibacter actinomycetemcomitans*, *Porphyromonas gingivalis*, and *Prevotella intermedia* were present. The patient had no history of recent dental extraction/oral surgery, radiotherapy, or chemotherapy.

Blood investigation: Calcium, potassium, phosphate, alkaline phosphatase, parathormone, 25‐hydroxy‐vitamin D3 (Calcidiol), and fibroblast growth factor 23 (FGF‐23) were all normal. There was slightly reduced beta‐Crosslaps (CTX) and osteocalcin. Based on the pretibial discolorations, marfanoid habitus, non‐attached gingiva, and prepubertal length growth spurt, molecular genetic testing was performed. The genetic analysis revealed pEDS, subtype c.890G > A, G297D. Anoctamin 5 was tested as negative.^10^, ^11^

## 3 RADIOLOGY

We performed cone beam computed tomography (CBCT; KaVo 3D eXam ConeBeam XG; KaVo Dental GmbH, Biberach/Riss, Germany) after the initial orthopantomography (OPT) (Orthophos XG, Sirona Dental Systems, Bensheim, Germany) to investigate the sclerotic apical changes on the OPT. As a result, we detected sclerotic apical changes using CBCT associated with all the teeth in Hounsfield unit and categorized these according to Jamdade and John.^6^

**FIGURE 1:**
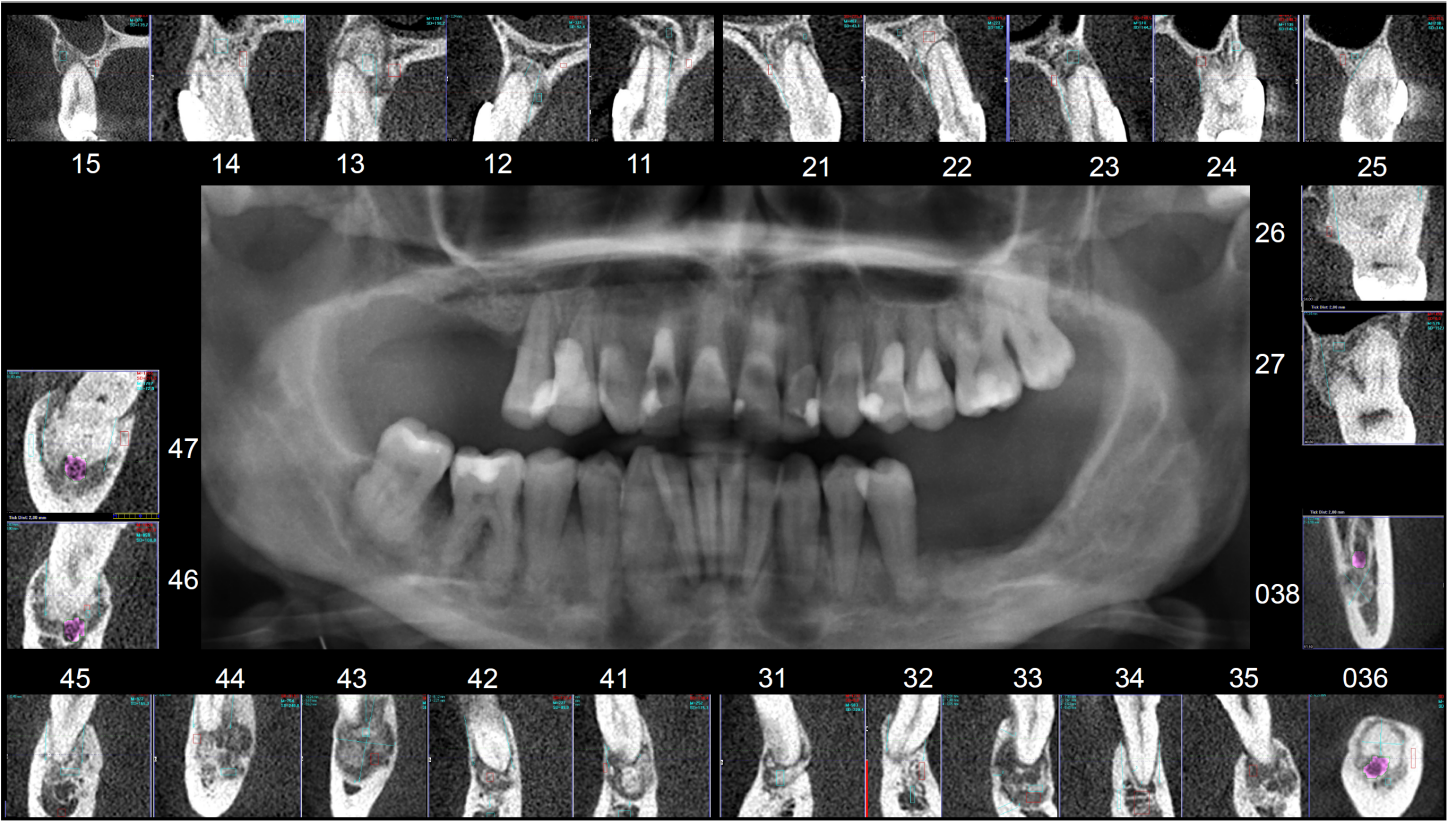
Center: CB‐CT‐ Orthopantomography‐Mode; Frame: Osteodensitometric detection by cross‐sectional images in the sagittal dimension from CB‐CT for each periapical region (CB‐CT‐O)

We compared the CBCT osteodensitometric results (CBCT‐O) to each other and assigned them according to the clinical‐radiological categories of maturation of periapical dysplasias.^12^, ^13^, ^14^ We performed radionuclide (BS) 2 h after an intravenous injection of technetium‐99 m methylene diphosphonate (^99^Tcm MDP; 520.0 MBq after 2 h; Pixel Size 2.1, SL 1880; SP 4.2 mm/sec) in the anteroposterior and lateral projections.^14^ We concluded and performed the diagnosis according to the bone scintigraphy [BS] and CBCT to exclude aggressive bone lesions.^13^ We verified both findings and correlated these with dental nerves and bone specimens^15^, ^16^, ^17^ (Figure 1).

The patient provided informed consent for an investigation using a technetium bone scan.

**FIGURE 2:**
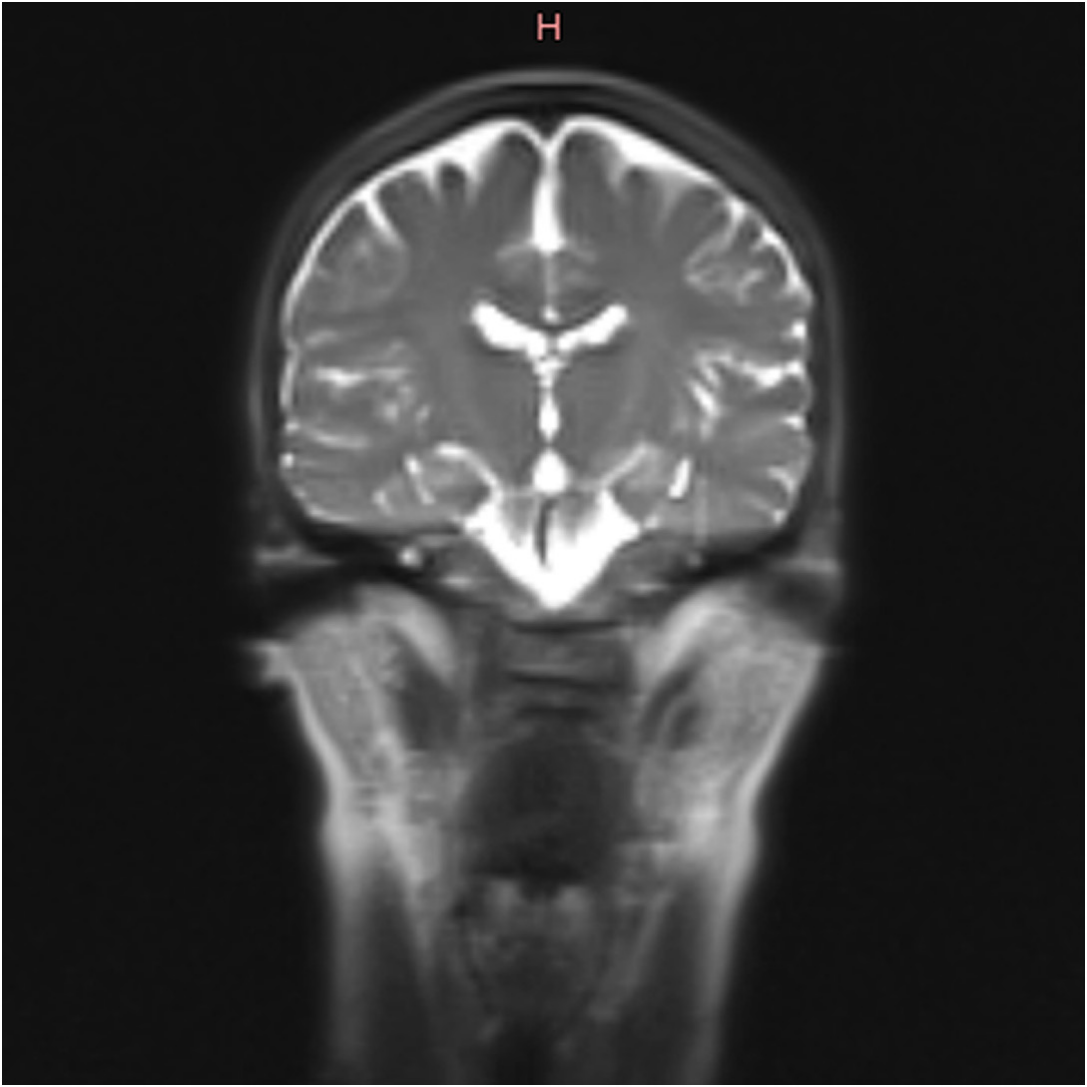
Cranial MR‐imaging shows confluent, symmetric periventricular white matter hyperintensities on T2‐weighted studies and slightly hypointensive signal relative to adjacent brain on T1‐weighted studies. Cortex and basal ganglia appeared to be normal in signal and morphology

**FIGURE 3:**
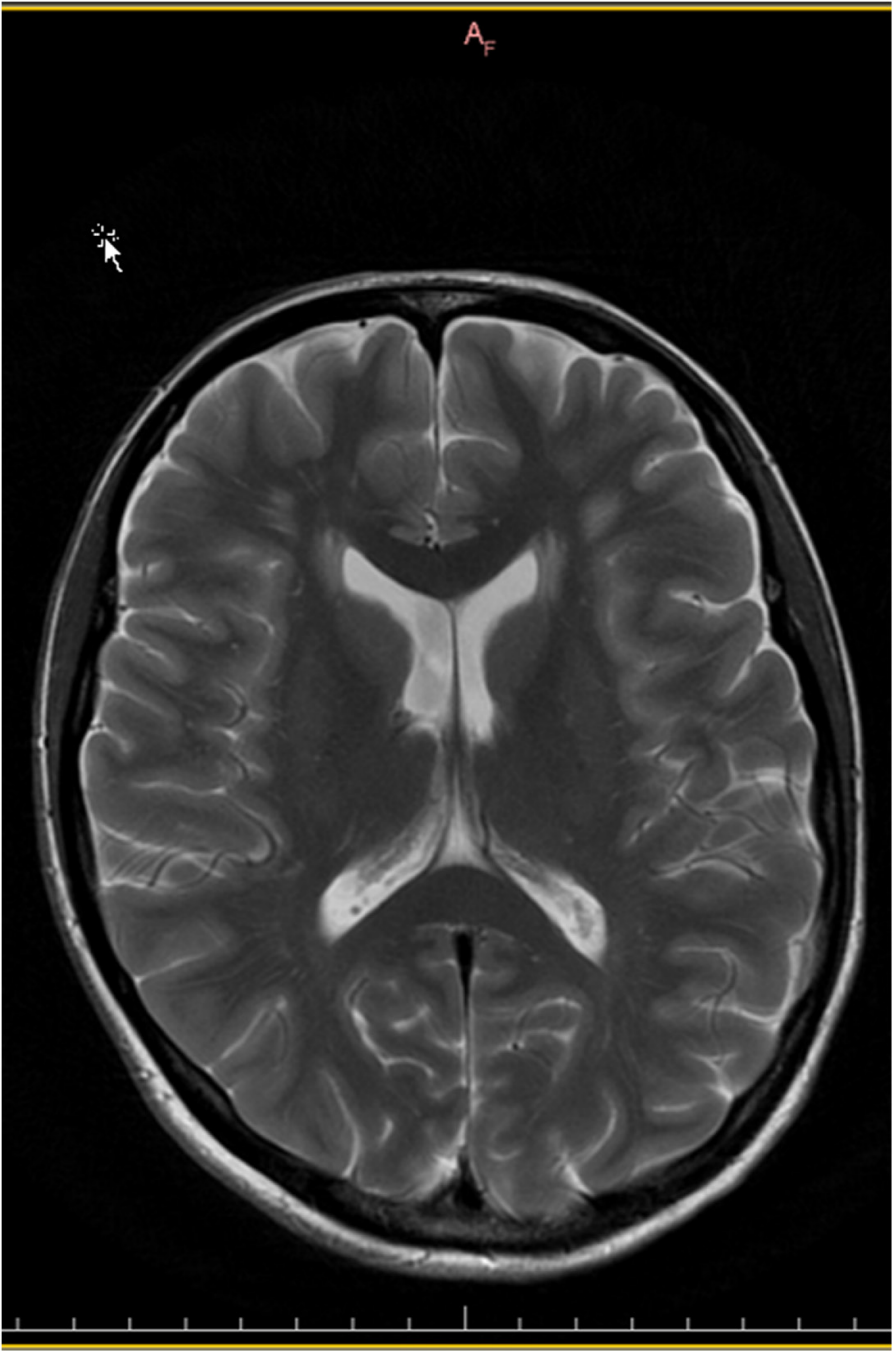
Cranial MR imaging shows symmetric periventricular white matter hyperintensities on T2‐weighted studies and slightly hypointensive signal relative to adjacent brain on T1‐weighted studies. No ventricular enlargement or mass effect was present

The magnetic resonance imaging (MRI) scan of the neurocranium revealed a medium‐sized, symmetrical ventricular system. The cerebral and cerebellar hemispheres were regular. Small‐spot confluent hyperintensities subcortically in the fluid‐attenuated inversion recovery (FLAIR) sequence were recognizable (Figures 2, and 3). Furthermore, the incidental findings included hypoplasia of the left frontal sinus and aplasia of the right frontal sinus.^18^

## 4 RESULTS

All of the periapical regions had radiological findings. We identified several areas in the upper jaw and the mandible with a nuclide enrichment in the scintigraphy. Therefore, we decided to separate the four groups of the ^99^Tcm uptake intensity as follows: No ^99^Tcm uptake (BSgrade: 0); 1: mild ^99^Tcm uptake (BSgrade: 1); 2: moderate ^99^Tcm uptake (BSgrade: 2); and 3: solid ^99^Tcm uptake (BSgrade: 3). The categories were summarized and compared to the CBCT findings. Based on the categories of maturation^8^ and the ^99^Tcm uptake, we found the following densities in the CBCT.

In regions 15, 11, 21, 23, 34, and 32: CBCT°1 (Grade 1): intraosseous ballooning, thinning of the cortical bone; and the beginning accumulation on the ^99^Tcm scintigraphy (BS grade: 1). In regions 12, 22, 38, 35, 33, and 31–45: CBCT°2 (Grade 2): apico‐central sclerosis and sclerotic margin (edging) to cancellous bone increasing accumulation on the ^99^Tcm scintigraphy (BS grade: 2). In regions 14, 13, 26, 27, and 46–47: CBCT°3 (Grade 3): sclerotic remodulation of the bone marrow and intense accumulation on the ^99^Tcm‐scintigraphy (BS grade: 3). Finally, in regions 36 and 37: CBCT°4 (Grade 4): periapical drumstick formation, hypercementosis, and decreasing accumulation on the ^99^Tcm ‐scintigraphy (BS grades: 1–2).

In regions 24 and 25, we did not find an accumulation of the ^99^Tcm in the scintigraphy, nor did we find it in the highly atrophic maxilla regions of 17 and 16. Morphological changes could be seen in the edentulous regions, but no technetium enrichment. No calcification of the pulp and no pulp stones were detected.

## 5 DISCUSSION

Fibro‐osseous lesions begin with a central focus (nidus) of mineralization, primarily at the apical portion of the teeth.^15^ In this location, there is the initial replacement of the spongy bone with fibrous tissue, and this is followed by increasing mineralization.^19^ CBCT osteodensitometry allows for the categorization of these changes and supports clinical monitoring.^20^ To determine the precise categorization and to separate these findings with different bone densities, the clinicopathological findings from hyperparathyroidism‐jaw tumor syndrome (HPT‐JT), gnathodiaphysial dysplasia (GDD), and familial gigantiform cementoma (FGC) can be considered.^21^, ^22^, ^23^, ^24^, ^25^

HPT‐JT is a genetic disorder associated with multiple parathyroid adenomas, renal tumors, and multiple ossifying fibromas. In a previous study, an investigation of blood did not reveal changes in the parathormone, nor did any co‐morbidity show evidence of HPT‐JT.^26^ GDD and FGC show phenotypical similarities, but patients with GDD often suffer from instability of the long bones and bone fractures. As there was no evidence of injuries in the patient of the present study, a physically active woman (BMI 21), we excluded this diagnosis.^27^ Similar to HPT‐JT and GDD, FGC is a systematic genetic disorder. In the present study, the biological mother, the deceased father's sister, and a cousin on the father's side all had genetic panel examinations that included pEDS, Anoctamin 5, HPT‐JT, and other bone‐related genetic disorders. However, these relatives showed no evidence of pEDS or FGC. Some researchers have suggested that familial florid cemento‐osseous dysplasia (COD) mimics FGC or represents different spectrums of the same clinicopathological process.^22^, ^23^, ^24^ Therefore, we excluded FGC by detecting Anoctamin 5 negative.^26^ Further research and genetic studies are required to improve the understanding and assess if the conditions are indeed related.

A few studies have reported periapical manifestations in classical EDS^27^ but not in pEDS, as recently reviewed by Kapferer‐Seebacher et al.^28^ Pulp calcification and pulp stones in classical EDS were first described by Selliseth in 1965^29^ and confirmed by others.^30^, ^31^, ^32^ In this case of pEDS, we did not find atypical pulp calcification in the remaining 24 teeth as described by Ferré et al.^32^ or Majorana and Facchetti^33^ for the classical EDS, but there was drumstick formation of the molars. This was similar to the presenting case of a 41‐year‐old woman described by Kapferer‐Seebacher et al. (2020),^2^ in which the authors reported that the calcification of the pulp was a common finding in cEDS, reported in 15 out of 17 individuals, and it was caused primarily by COL5A1 or COL5A2. In addition, irritations, decay, or chronic inflammation may induce pulp calcification. In the present case, the initial lesion appeared to arise from the periapical vascular bundle from which the nidus‐like periapical mineralization was derived as a secondary reaction. Diagnostically, pulp calcification must be differentiated from periapical cementum dysplasia. The authors hypothesized a morphopathological aberration of the nerve‐vascular bundle. This is typically located both at the foramen apicale and in the tooth itself. It seems, at least in consideration of the CBCT osteodensitometry evaluation, that the periapical cementum dysplasia, which progresses in different stages, ossifies focally or eventually scleroses. pEDS typically leads to premature tooth loss. The present case may show tooth preservation and dentomaxillary development as a consequence of intensive dental hygiene due to monthly visits by a dental hygienist. The biopsies revealed histological vascular proliferation in addition to bone sclerosis and fibrosis, and the biopsies confirmed the CBCT‐O findings.

Leukoencephalopathy was previously reported by Spranger in 1996^34^ to be combined with periodontal EDS. Kapferer Seeberger in 2018^35^ reported an association of pEDS with leukoencephylopathy in eight patients from two different families. Exome sequencing revealed C1R mutations c.926G > T and c.149_150TC > AT in these families. While one eight‐year‐old child had no radiographic white matter changes, the neuroradiological signs increased with age. In summary, the neurological complaints were considered to be disproportionate to the radiological findings and were similar in appearance to the MRI changes described for CADASIL, CARASIL, or CARASAL.^36^, ^37^, ^38^

It remains unclear whether the extent of the periapical inflammation in the sense of microangiopathic processes can be understood. As such, we assumed that vascular changes could be a trigger for periapical cement dysplasia. The same consideration of a microangiopathic genesis would also make sense concerning the deterioration of vision, with unfortunately no ability to be detected or objectified using an ophthalmological investigation.^34^, ^35^, ^36^, ^37^, ^38^, ^39^, ^40^

## AUTHOR CONTRIBUTIONS

MN contributed to the interdisciplinary planning, performing the surgical interventions, documentation of the case, and writing and editing of the manuscript. MHN contributed to the genetic counseling and writing of the manuscript. CM contributed to the pathologic examination and writing of the manuscript. BK contributed to the surgical and cardiovascular counseling and writing of the manuscript. GL contributed to counseling in oral surgery and writing and editing of the manuscript. MK contributed to the ophthalmologic examination and writing of the manuscript. All authors contributed to the study's conception and design; commented on previous versions of the manuscript and read and approved the final manuscript. We also thank LetPub (www.letpub.com) for its linguistic assistance during the preparation of this manuscript.

## ACKNOWLEDGMENTS

The authors declare that they have no known competing financial interests or personal relationships that could have appeared to influence the work reported in this paper.

## FUNDING INFORMATION

This research did not receive any specific grant from funding agencies in the public, commercial, or not‐for‐profit sectors.

## ETHICAL APPROVAL

The authors ensure that the work described was conducted following the Code of Ethics of the World Medical Association (Declaration of Helsinki). This manuscript is in line with the Recommendations for the Conduct, Reporting, Editing, and Publication of Scholarly Work in Medical Journals and aims to include representative human populations (sex, age, and ethnicity) as per those recommendations. This article does not contain any experiments that used human or animal subjects.

## CONSENT

Written informed consent was obtained from the patient to publish this report in accordance with the journal's patient consent policy.

## Data Availability

The data that support the findings of this study are available from the corresponding author upon reasonable request.
